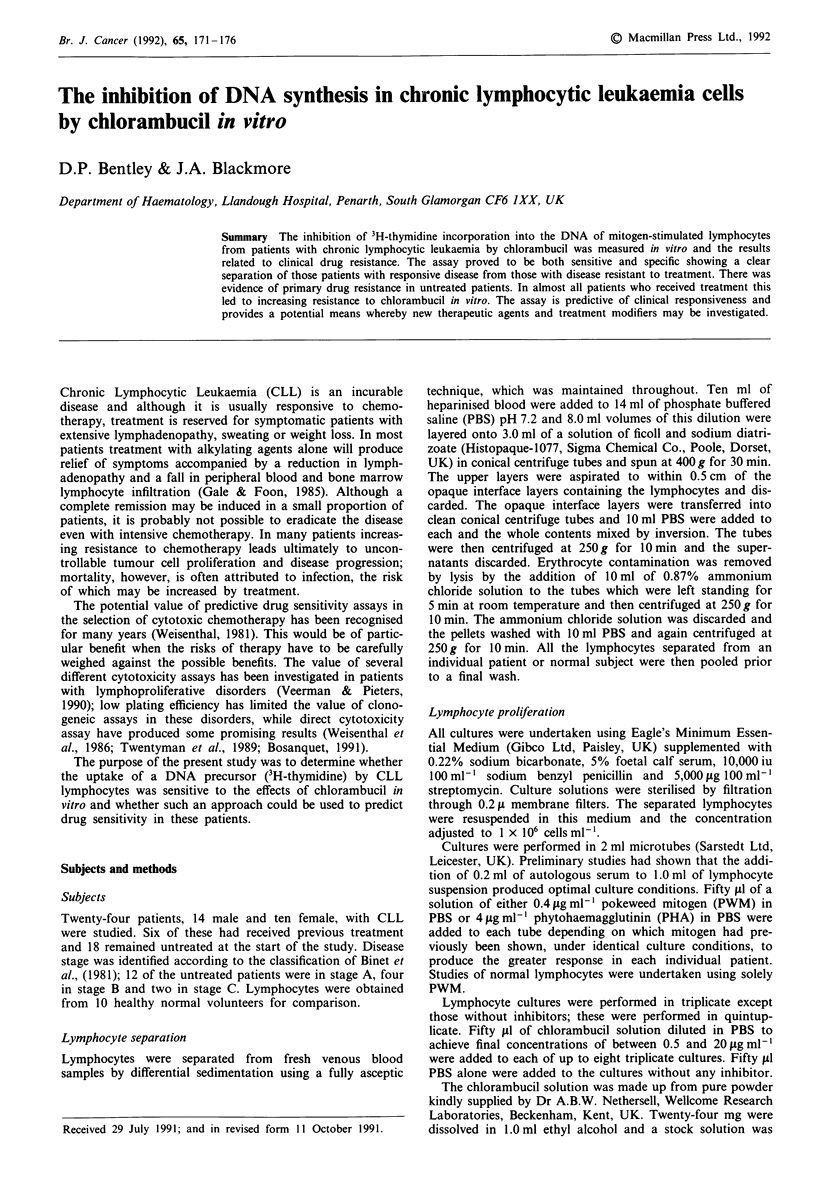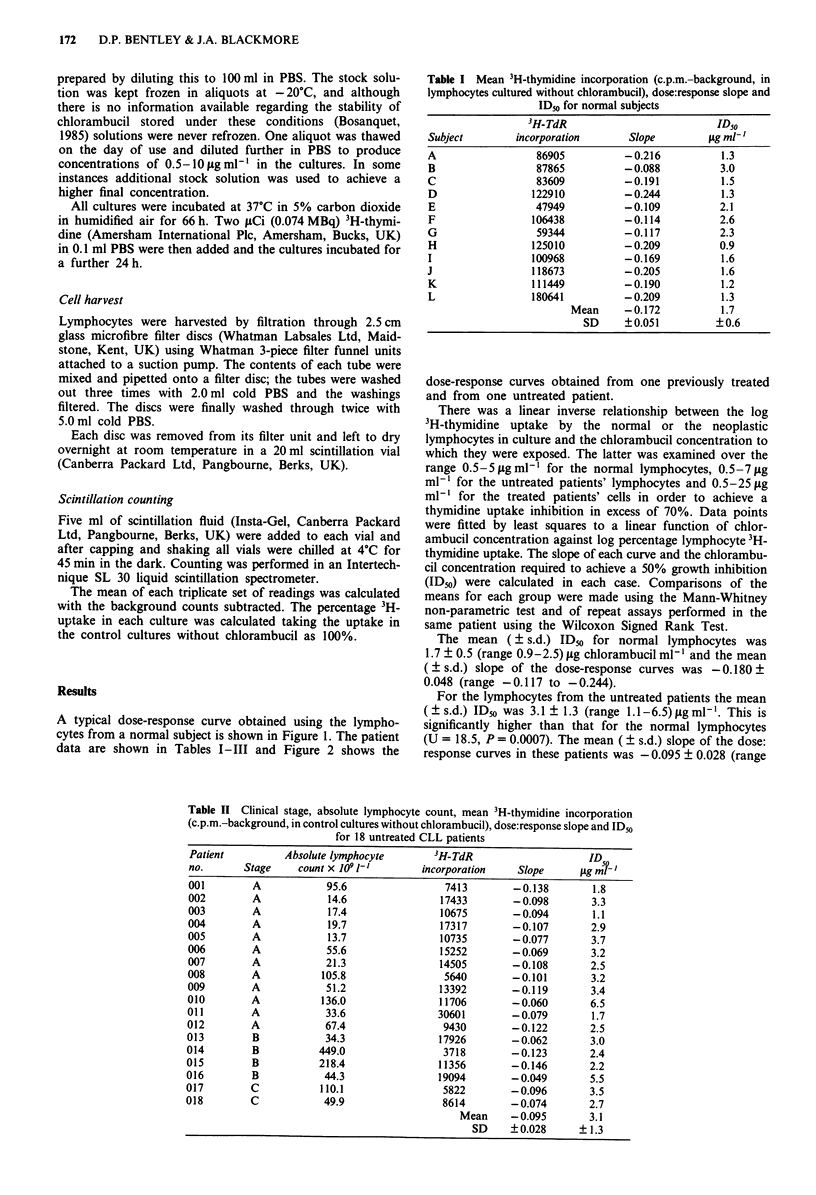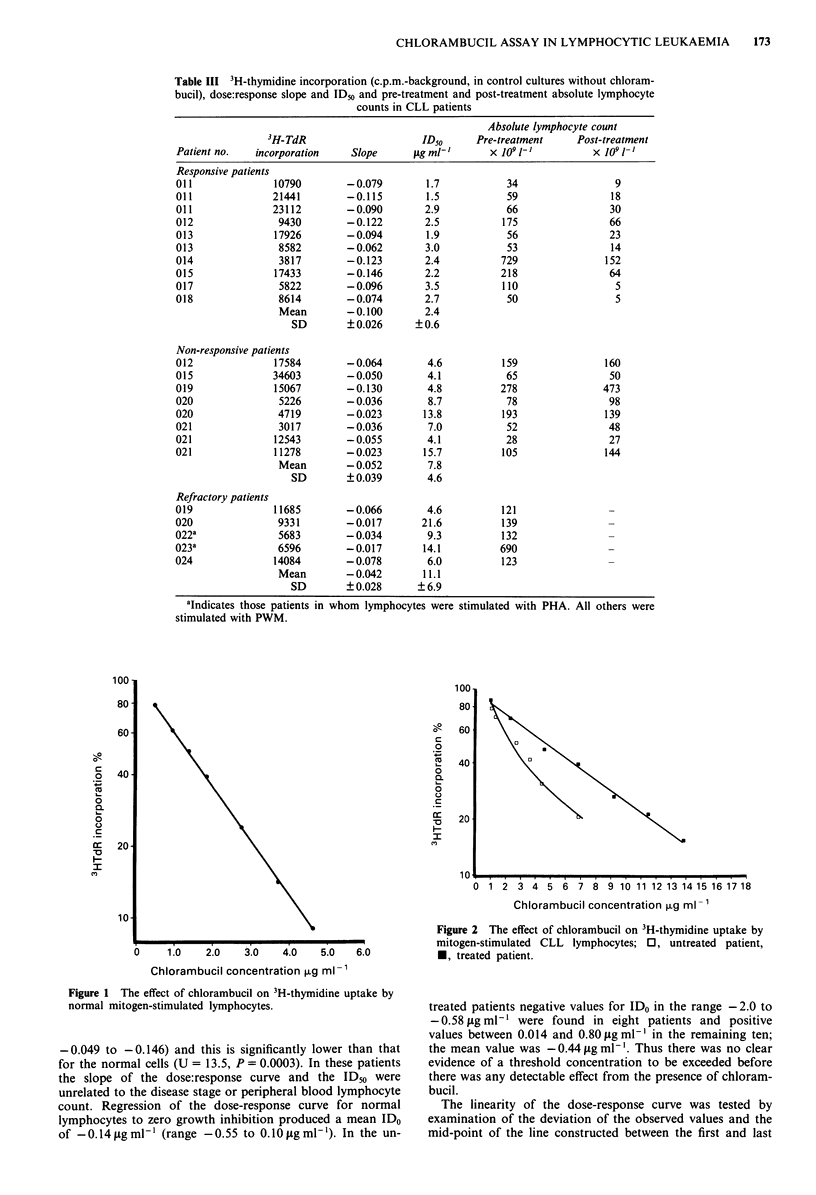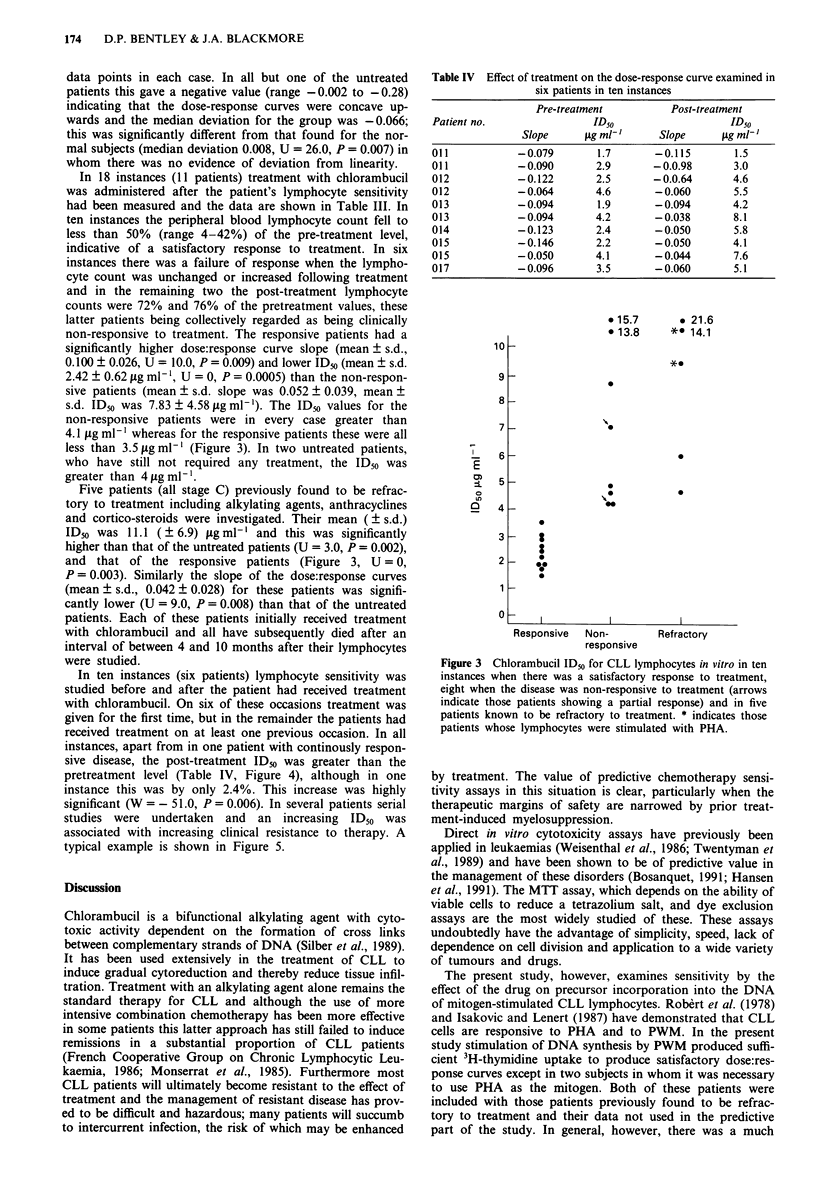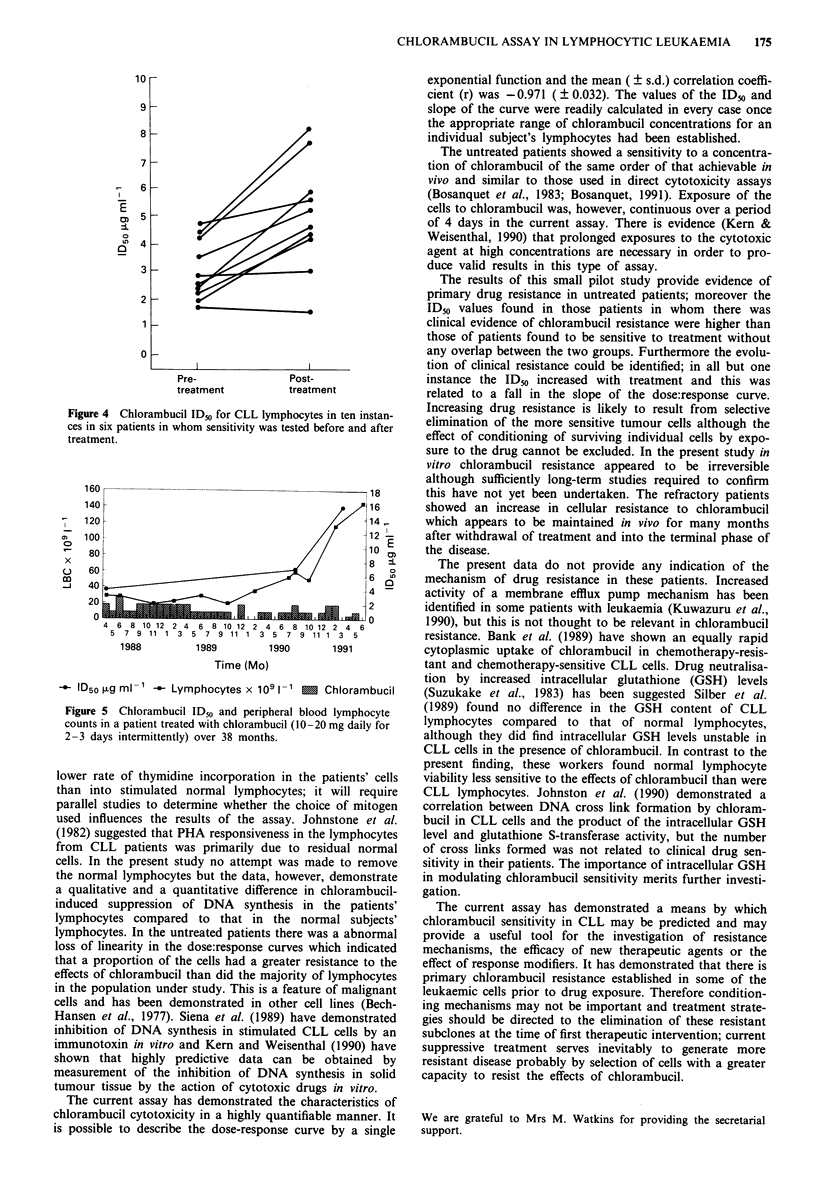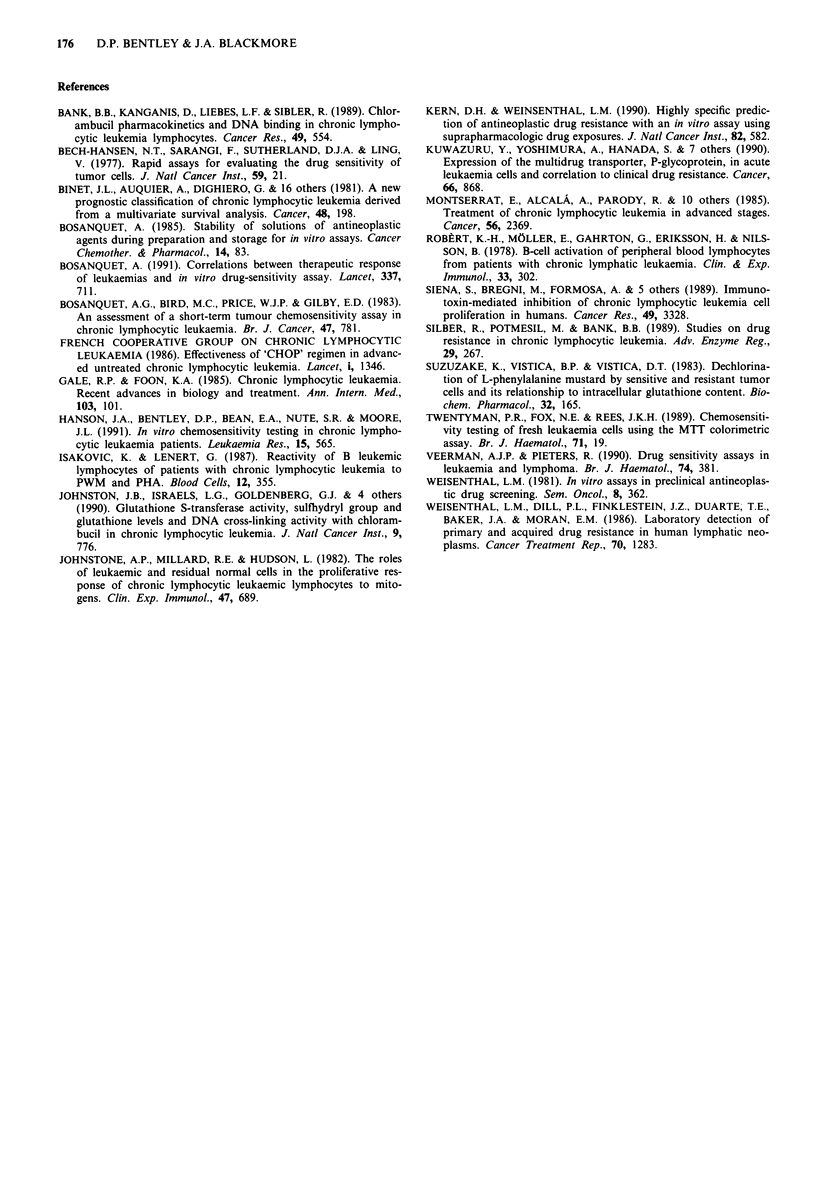# The inhibition of DNA synthesis in chronic lymphocytic leukaemia cells by chlorambucil in vitro.

**DOI:** 10.1038/bjc.1992.36

**Published:** 1992-02

**Authors:** D. P. Bentley, J. A. Blackmore

**Affiliations:** Department of Haematology, Llandough Hospital, Penarth, South Glamorgan, UK.

## Abstract

The inhibition of 3H-thymidine incorporation into the DNA of mitogen-stimulated lymphocytes from patients with chronic lymphocytic leukaemia by chlorambucil was measured in vitro and the results related to clinical drug resistance. The assay proved to be both sensitive and specific showing a clear separation of those patients with responsive disease from those with disease resistant to treatment. There was evidence of primary drug resistance in untreated patients. In almost all patients who received treatment this led to increasing resistance to chlorambucil in vitro. The assay is predictive of clinical responsiveness and provides a potential means whereby new therapeutic agents and treatment modifiers may be investigated.


Br. J. Cancer (1992), 65, 171–176 © Macmillan Press Ltd., 1992

### The inhibition of DNA synthesis in chronic lymphocytic leukaemia cells by chlorambucil *in vitro*

D.P. Bentley & J.A. Blackmore

*Department of Haematology, Llandough Hospital, Penarth, South Glamorgan CF6 1XX, UK*

**Summary** The inhibition of $^3$H-thymidine incorporation into the DNA of mitogen-stimulated lymphocytes from patients with chronic lymphocytic leukaemia by chlorambucil was measured *in vitro* and the results related to clinical drug resistance. The assay proved to be both sensitive and specific showing a clear separation of those patients with responsive disease from those with disease resistant to treatment. There was evidence of primary drug resistance in untreated patients. In almost all patients who received treatment this led to increasing resistance to chlorambucil *in vitro*. The assay is predictive of clinical responsiveness and provides a potential means whereby new therapeutic agents and treatment modifiers may be investigated.

Chronic Lymphocytic Leukaemia (CLL) is an incurable disease and although it is usually responsive to chemotherapy, treatment is reserved for symptomatic patients with extensive lymphadenopathy, sweating or weight loss. In most patients treatment with alkylating agents alone will produce relief of symptoms accompanied by a reduction in lymphadenopathy and a fall in peripheral blood and bone marrow lymphocyte infiltration (Gale & Foon, 1985). Although a complete remission may be induced in a small proportion of patients, it is probably not possible to eradicate the disease even with intensive chemotherapy. In many patients increasing resistance to chemotherapy leads ultimately to uncontrollable tumour cell proliferation and disease progression; mortality, however, is often attributed to infection, the risk of which may be increased by treatment.

The potential value of predictive drug sensitivity assays in the selection of cytotoxic chemotherapy has been recognised for many years (Weisenthal, 1981). This would be of particular benefit when the risks of therapy have to be carefully weighed against the possible benefits. The value of several different cytotoxicity assays has been investigated in patients with lymphoproliferative disorders (Veerman & Pieters, 1990); low plating efficiency has limited the value of clonogeneic assays in these disorders, while direct cytotoxicity assay have produced some promising results (Weisenthal *et al.*, 1986; Twentyman *et al.*, 1989; Bosanquet, 1991).

The purpose of the present study was to determine whether the uptake of a DNA precursor ($^3$H-thymidine) by CLL lymphocytes was sensitive to the effects of chlorambucil *in vitro* and whether such an approach could be used to predict drug sensitivity in these patients.

### Subjects and methods

### *Subjects*

Twenty-four patients, 14 male and ten female, with CLL were studied. Six of these had received previous treatment and 18 remained untreated at the start of the study. Disease stage was identified according to the classification of Binet *et al.*, (1981); 12 of the untreated patients were in stage A, four in stage B and two in stage C. Lymphocytes were obtained from 10 healthy normal volunteers for comparison.

### *Lymphocyte separation*

Lymphocytes were separated from fresh venous blood samples by differential sedimentation using a fully asceptic technique, which was maintained throughout. Ten ml of heparinised blood were added to 14 ml of phosphate buffered saline (PBS) pH 7.2 and 8.0 ml volumes of this dilution were layered onto 3.0 ml of a solution of ficoll and sodium diatrizoate (Histopaque-1077, Sigma Chemical Co., Poole, Dorset, UK) in conical centrifuge tubes and spun at 400 *g* for 30 min. The upper layers were aspirated to within 0.5 cm of the opaque interface layers containing the lymphocytes and discarded. The opaque interface layers were transferred into clean conical centrifuge tubes and 10 ml PBS were added to each and the whole contents mixed by inversion. The tubes were then centrifuged at 250 *g* for 10 min and the supernatants discarded. Erythrocyte contamination was removed by lysis by the addition of 10 ml of 0.87% ammonium chloride solution to the tubes which were left standing for 5 min at room temperature and then centrifuged at 250 *g* for 10 min. The ammonium chloride solution was discarded and the pellets washed with 10 ml PBS and again centrifuged at 250 *g* for 10 min. All the lymphocytes separated from an individual patient or normal subject were then pooled prior to a final wash.

### *Lymphocyte proliferation*

All cultures were undertaken using Eagle's Minimum Essential Medium (Gibco Ltd, Paisley, UK) supplemented with 0.22% sodium bicarbonate, 5% foetal calf serum, 10,000 iu 100 ml$^{-1}$ sodium benzyl penicillin and 5,000 $\mu$g 100 ml$^{-1}$ streptomycin. Culture solutions were sterilised by filtration through 0.2 $\mu$ membrane filters. The separated lymphocytes were resuspended in this medium and the concentration adjusted to $1 \times 10^6$ cells ml$^{-1}$.

Cultures were performed in 2 ml microtubes (Sarstedt Ltd, Leicester, UK). Preliminary studies had shown that the addition of 0.2 ml of autologous serum to 1.0 ml of lymphocyte suspension produced optimal culture conditions. Fifty $\mu$l of a solution of either 0.4 $\mu$g ml$^{-1}$ pokeweed mitogen (PWM) in PBS or 4 $\mu$g ml$^{-1}$ phytohaemagglutinin (PHA) in PBS were added to each tube depending on which mitogen had previously been shown, under identical culture conditions, to produce the greater response in each individual patient. Studies of normal lymphocytes were undertaken using solely PWM.

Lymphocyte cultures were performed in triplicate except those without inhibitors; these were performed in quintuplicate. Fifty $\mu$l of chlorambucil solution diluted in PBS to achieve final concentrations of between 0.5 and 20 $\mu$g ml$^{-1}$ were added to each of up to eight triplicate cultures. Fifty $\mu$l PBS alone were added to the cultures without any inhibitor.

The chlorambucil solution was made up from pure powder kindly supplied by Dr A.B.W. Nethersell, Wellcome Research Laboratories, Beckenham, Kent, UK. Twenty-four mg were dissolved in 1.0 ml ethyl alcohol and a stock solution was

Received 29 July 1991; and in revised form 11 October 1991.

prepared by diluting this to 100 ml in PBS. The stock solution was kept frozen in aliquots at −20°C, and although there is no information available regarding the stability of chlorambucil stored under these conditions (Bosanquet, 1985) solutions were never refrozen. One aliquot was thawed on the day of use and diluted further in PBS to produce concentrations of 0.5–10 $\mu g\,ml^{-1}$ in the cultures. In some instances additional stock solution was used to achieve a higher final concentration.

All cultures were incubated at 37°C in 5% carbon dioxide in humidified air for 66 h. Two $\mu Ci$ (0.074 MBq) $^{3}H$-thymidine (Amersham International Plc, Amersham, Bucks, UK) in 0.1 ml PBS were then added and the cultures incubated for a further 24 h.

*Cell harvest*

Lymphocytes were harvested by filtration through 2.5 cm glass microfibre filter discs (Whatman Labsales Ltd, Maidstone, Kent, UK) using Whatman 3-piece filter funnel units attached to a suction pump. The contents of each tube were mixed and pipetted onto a filter disc; the tubes were washed out three times with 2.0 ml cold PBS and the washings filtered. The discs were finally washed through twice with 5.0 ml cold PBS.

Each disc was removed from its filter unit and left to dry overnight at room temperature in a 20 ml scintillation vial (Canberra Packard Ltd, Pangbourne, Berks, UK).

*Scintillation counting*

Five ml of scintillation fluid (Insta-Gel, Canberra Packard Ltd, Pangbourne, Berks, UK) were added to each vial and after capping and shaking all vials were chilled at 4°C for 45 min in the dark. Counting was performed in an Intertechnique SL 30 liquid scintillation spectrometer.

The mean of each triplicate set of readings was calculated with the background counts subtracted. The percentage $^{3}H$-uptake in each culture was calculated taking the uptake in the control cultures without chlorambucil as 100%.

### Results

A typical dose-response curve obtained using the lymphocytes from a normal subject is shown in Figure 1. The patient data are shown in Tables I–III and Figure 2 shows the

**Table I** Mean $^{3}H$-thymidine incorporation (c.p.m.–background, in lymphocytes cultured without chlorambucil), dose:response slope and $ID_{50}$ for normal subjects

| *Subject* | *$^{3}H$-TdR incorporation* | *Slope* | *$ID_{50}$ $\mu g\,ml^{-1}$* |
|---|---|---|---|
| A | 86905 | −0.216 | 1.3 |
| B | 87865 | −0.088 | 3.0 |
| C | 83609 | −0.191 | 1.5 |
| D | 122910 | −0.244 | 1.3 |
| E | 47949 | −0.109 | 2.1 |
| F | 106438 | −0.114 | 2.6 |
| G | 59344 | −0.117 | 2.3 |
| H | 125010 | −0.209 | 0.9 |
| I | 100968 | −0.169 | 1.6 |
| J | 118673 | −0.205 | 1.6 |
| K | 111449 | −0.190 | 1.2 |
| L | 180641 | −0.209 | 1.3 |
| | Mean | −0.172 | 1.7 |
| | SD | ±0.051 | ±0.6 |

dose-response curves obtained from one previously treated and from one untreated patient.

There was a linear inverse relationship between the log $^{3}H$-thymidine uptake by the normal or the neoplastic lymphocytes in culture and the chlorambucil concentration to which they were exposed. The latter was examined over the range 0.5–5 $\mu g\,ml^{-1}$ for the normal lymphocytes, 0.5–7 $\mu g\,ml^{-1}$ for the untreated patients' lymphocytes and 0.5–25 $\mu g\,ml^{-1}$ for the treated patients' cells in order to achieve a thymidine uptake inhibition in excess of 70%. Data points were fitted by least squares to a linear function of chlorambucil concentration against log percentage lymphocyte $^{3}H$-thymidine uptake. The slope of each curve and the chlorambucil concentration required to achieve a 50% growth inhibition ($ID_{50}$) were calculated in each case. Comparisons of the means for each group were made using the Mann-Whitney non-parametric test and of repeat assays performed in the same patient using the Wilcoxon Signed Rank Test.

The mean (± s.d.) $ID_{50}$ for normal lymphocytes was 1.7 ± 0.5 (range 0.9–2.5) $\mu g$ chlorambucil $ml^{-1}$ and the mean (± s.d.) slope of the dose-response curves was −0.180 ± 0.048 (range −0.117 to −0.244).

For the lymphocytes from the untreated patients the mean (± s.d.) $ID_{50}$ was 3.1 ± 1.3 (range 1.1–6.5) $\mu g\,ml^{-1}$. This is significantly higher than that for the normal lymphocytes ($U = 18.5$, $P = 0.0007$). The mean (± s.d.) slope of the dose: response curves in these patients was −0.095 ± 0.028 (range

**Table II** Clinical stage, absolute lymphocyte count, mean $^{3}H$-thymidine incorporation (c.p.m.–background, in control cultures without chlorambucil), dose:response slope and $ID_{50}$ for 18 untreated CLL patients

| *Patient no.* | *Stage* | *Absolute lymphocyte count × $10^{9}\,l^{-1}$* | *$^{3}H$-TdR incorporation* | *Slope* | *$ID_{50}$ $\mu g\,ml^{-1}$* |
|---|---|---|---|---|---|
| 001 | A | 95.6 | 7413 | −0.138 | 1.8 |
| 002 | A | 14.6 | 17433 | −0.098 | 3.3 |
| 003 | A | 17.4 | 10675 | −0.094 | 1.1 |
| 004 | A | 19.7 | 17317 | −0.107 | 2.9 |
| 005 | A | 13.7 | 10735 | −0.077 | 3.7 |
| 006 | A | 55.6 | 15252 | −0.069 | 3.2 |
| 007 | A | 21.3 | 14505 | −0.108 | 2.5 |
| 008 | A | 105.8 | 5640 | −0.101 | 3.2 |
| 009 | A | 51.2 | 13392 | −0.119 | 3.4 |
| 010 | A | 136.0 | 11706 | −0.060 | 6.5 |
| 011 | A | 33.6 | 30601 | −0.079 | 1.7 |
| 012 | A | 67.4 | 9430 | −0.122 | 2.5 |
| 013 | B | 34.3 | 17926 | −0.062 | 3.0 |
| 014 | B | 449.0 | 3718 | −0.123 | 2.4 |
| 015 | B | 218.4 | 11356 | −0.146 | 2.2 |
| 016 | B | 44.3 | 19094 | −0.049 | 5.5 |
| 017 | C | 110.1 | 5822 | −0.096 | 3.5 |
| 018 | C | 49.9 | 8614 | −0.074 | 2.7 |
| | | | Mean | −0.095 | 3.1 |
| | | | SD | ±0.028 | ±1.3 |

**Table III** $^3$H-thymidine incorporation (c.p.m.-background, in control cultures without chlorambucil), dose:response slope and $ID_{50}$ and pre-treatment and post-treatment absolute lymphocyte counts in CLL patients

| Patient no. | $^3$H-TdR incorporation | Slope | $ID_{50}$ μg ml$^{-1}$ | Absolute lymphocyte count: Pre-treatment × $10^9$ l$^{-1}$ | Absolute lymphocyte count: Post-treatment × $10^9$ l$^{-1}$ |
|---|---|---|---|---|---|
| *Responsive patients* | | | | | |
| 011 | 10790 | −0.079 | 1.7 | 34 | 9 |
| 011 | 21441 | −0.115 | 1.5 | 59 | 18 |
| 011 | 23112 | −0.090 | 2.9 | 66 | 30 |
| 012 | 9430 | −0.122 | 2.5 | 175 | 66 |
| 013 | 17926 | −0.094 | 1.9 | 56 | 23 |
| 013 | 8582 | −0.062 | 3.0 | 53 | 14 |
| 014 | 3817 | −0.123 | 2.4 | 729 | 152 |
| 015 | 17433 | −0.146 | 2.2 | 218 | 64 |
| 017 | 5822 | −0.096 | 3.5 | 110 | 5 |
| 018 | 8614 | −0.074 | 2.7 | 50 | 5 |
| | Mean | −0.100 | 2.4 | | |
| | SD | ±0.026 | ±0.6 | | |
| *Non-responsive patients* | | | | | |
| 012 | 17584 | −0.064 | 4.6 | 159 | 160 |
| 015 | 34603 | −0.050 | 4.1 | 65 | 50 |
| 019 | 15067 | −0.130 | 4.8 | 278 | 473 |
| 020 | 5226 | −0.036 | 8.7 | 78 | 98 |
| 020 | 4719 | −0.023 | 13.8 | 193 | 139 |
| 021 | 3017 | −0.036 | 7.0 | 52 | 48 |
| 021 | 12543 | −0.055 | 4.1 | 28 | 27 |
| 021 | 11278 | −0.023 | 15.7 | 105 | 144 |
| | Mean | −0.052 | 7.8 | | |
| | SD | ±0.039 | 4.6 | | |
| *Refractory patients* | | | | | |
| 019 | 11685 | −0.066 | 4.6 | 121 | – |
| 020 | 9331 | −0.017 | 21.6 | 139 | – |
| 022[a] | 5683 | −0.034 | 9.3 | 132 | – |
| 023[a] | 6596 | −0.017 | 14.1 | 690 | – |
| 024 | 14084 | −0.078 | 6.0 | 123 | – |
| | Mean | −0.042 | 11.1 | | |
| | SD | ±0.028 | ±6.9 | | |

[a]Indicates those patients in whom lymphocytes were stimulated with PHA. All others were stimulated with PWM.

**Figure 1** The effect of chlorambucil on $^3$H-thymidine uptake by normal mitogen-stimulated lymphocytes.

**Figure 2** The effect of chlorambucil on $^3$H-thymidine uptake by mitogen-stimulated CLL lymphocytes; □, untreated patient, ■, treated patient.

−0.049 to −0.146) and this is significantly lower than that for the normal cells ($U = 13.5$, $P = 0.0003$). In these patients the slope of the dose:response curve and the $ID_{50}$ were unrelated to the disease stage or peripheral blood lymphocyte count. Regression of the dose-response curve for normal lymphocytes to zero growth inhibition produced a mean $ID_0$ of −0.14 μg ml$^{-1}$ (range −0.55 to 0.10 μg ml$^{-1}$). In the untreated patients negative values for $ID_0$ in the range −2.0 to −0.58 μg ml$^{-1}$ were found in eight patients and positive values between 0.014 and 0.80 μg ml$^{-1}$ in the remaining ten; the mean value was −0.44 μg ml$^{-1}$. Thus there was no clear evidence of a threshold concentration to be exceeded before there was any detectable effect from the presence of chlorambucil.

The linearity of the dose-response curve was tested by examination of the deviation of the observed values and the mid-point of the line constructed between the first and last data points in each case. In all but one of the untreated patients this gave a negative value (range −0.002 to −0.28) indicating that the dose-response curves were concave upwards and the median deviation for the group was −0.066; this was significantly different from that found for the normal subjects (median deviation 0.008, U = 26.0, $P = 0.007$) in whom there was no evidence of deviation from linearity.

In 18 instances (11 patients) treatment with chlorambucil was administered after the patient's lymphocyte sensitivity had been measured and the data are shown in Table III. In ten instances the peripheral blood lymphocyte count fell to less than 50% (range 4–42%) of the pre-treatment level, indicative of a satisfactory response to treatment. In six instances there was a failure of response when the lymphocyte count was unchanged or increased following treatment and in the remaining two the post-treatment lymphocyte counts were 72% and 76% of the pretreatment values, these latter patients being collectively regarded as being clinically non-responsive to treatment. The responsive patients had a significantly higher dose:response curve slope (mean ± s.d., 0.100 ± 0.026, U = 10.0, $P = 0.009$) and lower $ID_{50}$ (mean ± s.d. 2.42 ± 0.62 $\mu g\ ml^{-1}$, U = 0, $P = 0.0005$) than the non-responsive patients (mean ± s.d. slope was 0.052 ± 0.039, mean ± s.d. $ID_{50}$ was 7.83 ± 4.58 $\mu g\ ml^{-1}$). The $ID_{50}$ values for the non-responsive patients were in every case greater than 4.1 $\mu g\ ml^{-1}$ whereas for the responsive patients these were all less than 3.5 $\mu g\ ml^{-1}$ (Figure 3). In two untreated patients, who have still not required any treatment, the $ID_{50}$ was greater than 4 $\mu g\ ml^{-1}$.

Five patients (all stage C) previously found to be refractory to treatment including alkylating agents, anthracyclines and cortico-steroids were investigated. Their mean (± s.d.) $ID_{50}$ was 11.1 (± 6.9) $\mu g\ ml^{-1}$ and this was significantly higher than that of the untreated patients (U = 3.0, $P = 0.002$), and that of the responsive patients (Figure 3, U = 0, $P = 0.003$). Similarly the slope of the dose:response curves (mean ± s.d., 0.042 ± 0.028) for these patients was significantly lower (U = 9.0, $P = 0.008$) than that of the untreated patients. Each of these patients initially received treatment with chlorambucil and all have subsequently died after an interval of between 4 and 10 months after their lymphocytes were studied.

In ten instances (six patients) lymphocyte sensitivity was studied before and after the patient had received treatment with chlorambucil. On six of these occasions treatment was given for the first time, but in the remainder the patients had received treatment on at least one previous occasion. In all instances, apart from in one patient with continously responsive disease, the post-treatment $ID_{50}$ was greater than the pretreatment level (Table IV, Figure 4), although in one instance this was by only 2.4%. This increase was highly significant (W = − 51.0, $P = 0.006$). In several patients serial studies were undertaken and an increasing $ID_{50}$ was associated with increasing clinical resistance to therapy. A typical example is shown in Figure 5.

**Table IV** Effect of treatment on the dose-response curve examined in six patients in ten instances

| Patient no. | Pre-treatment Slope | Pre-treatment $ID_{50}$ $\mu g\ ml^{-1}$ | Post-treatment Slope | Post-treatment $ID_{50}$ $\mu g\ ml^{-1}$ |
|---|---|---|---|---|
| 011 | −0.079 | 1.7 | −0.115 | 1.5 |
| 011 | −0.090 | 2.9 | −0.0.98 | 3.0 |
| 012 | −0.122 | 2.5 | −0.0.64 | 4.6 |
| 012 | −0.064 | 4.6 | −0.060 | 5.5 |
| 013 | −0.094 | 1.9 | −0.094 | 4.2 |
| 013 | −0.094 | 4.2 | −0.038 | 8.1 |
| 014 | −0.123 | 2.4 | −0.050 | 5.8 |
| 015 | −0.146 | 2.2 | −0.050 | 4.1 |
| 015 | −0.050 | 4.1 | −0.044 | 7.6 |
| 017 | −0.096 | 3.5 | −0.060 | 5.1 |

**Figure 3** Chlorambucil $ID_{50}$ for CLL lymphocytes *in vitro* in ten instances when there was a satisfactory response to treatment, eight when the disease was non-responsive to treatment (arrows indicate those patients showing a partial response) and in five patients known to be refractory to treatment. * indicates those patients whose lymphocytes were stimulated with PHA.

### Discussion

Chlorambucil is a bifunctional alkylating agent with cytotoxic activity dependent on the formation of cross links between complementary strands of DNA (Silber *et al.*, 1989). It has been used extensively in the treatment of CLL to induce gradual cytoreduction and thereby reduce tissue infiltration. Treatment with an alkylating agent alone remains the standard therapy for CLL and although the use of more intensive combination chemotherapy has been more effective in some patients this latter approach has still failed to induce remissions in a substantial proportion of CLL patients (French Cooperative Group on Chronic Lymphocytic Leukaemia, 1986; Monserrat *et al.*, 1985). Furthermore most CLL patients will ultimately become resistant to the effect of treatment and the management of resistant disease has proved to be difficult and hazardous; many patients will succumb to intercurrent infection, the risk of which may be enhanced by treatment. The value of predictive chemotherapy sensitivity assays in this situation is clear, particularly when the therapeutic margins of safety are narrowed by prior treatment-induced myelosuppression.

Direct *in vitro* cytotoxicity assays have previously been applied in leukaemias (Weisenthal *et al.*, 1986; Twentyman *et al.*, 1989) and have been shown to be of predictive value in the management of these disorders (Bosanquet, 1991; Hansen *et al.*, 1991). The MTT assay, which depends on the ability of viable cells to reduce a tetrazolium salt, and dye exclusion assays are the most widely studied of these. These assays undoubtedly have the advantage of simplicity, speed, lack of dependence on cell division and application to a wide variety of tumours and drugs.

The present study, however, examines sensitivity by the effect of the drug on precursor incorporation into the DNA of mitogen-stimulated CLL lymphocytes. Robèrt *et al.* (1978) and Isakovic and Lenert (1987) have demonstrated that CLL cells are responsive to PHA and to PWM. In the present study stimulation of DNA synthesis by PWM produced sufficient $^{3}H$-thymidine uptake to produce satisfactory dose:response curves except in two subjects in whom it was necessary to use PHA as the mitogen. Both of these patients were included with those patients previously found to be refractory to treatment and their data not used in the predictive part of the study. In general, however, there was a much

**Figure 4** Chlorambucil $ID_{50}$ for CLL lymphocytes in ten instances in six patients in whom sensitivity was tested before and after treatment.

**Figure 5** Chlorambucil $ID_{50}$ and peripheral blood lymphocyte counts in a patient treated with chlorambucil (10–20 mg daily for 2–3 days intermittently) over 38 months.

lower rate of thymidine incorporation in the patients' cells than into stimulated normal lymphocytes; it will require parallel studies to determine whether the choice of mitogen used influences the results of the assay. Johnstone *et al.* (1982) suggested that PHA responsiveness in the lymphocytes from CLL patients was primarily due to residual normal cells. In the present study no attempt was made to remove the normal lymphocytes but the data, however, demonstrate a qualitative and a quantitative difference in chlorambucil-induced suppression of DNA synthesis in the patients' lymphocytes compared to that in the normal subjects' lymphocytes. In the untreated patients there was a abnormal loss of linearity in the dose:response curves which indicated that a proportion of the cells had a greater resistance to the effects of chlorambucil than did the majority of lymphocytes in the population under study. This is a feature of malignant cells and has been demonstrated in other cell lines (Bech-Hansen *et al.*, 1977). Siena *et al.* (1989) have demonstrated inhibition of DNA synthesis in stimulated CLL cells by an immunotoxin *in vitro* and Kern and Weisenthal (1990) have shown that highly predictive data can be obtained by measurement of the inhibition of DNA synthesis in solid tumour tissue by the action of cytotoxic drugs *in vitro*.

The current assay has demonstrated the characteristics of chlorambucil cytotoxicity in a highly quantifiable manner. It is possible to describe the dose-response curve by a single exponential function and the mean ($\pm$ s.d.) correlation coefficient (r) was $-0.971$ ($\pm 0.032$). The values of the $ID_{50}$ and slope of the curve were readily calculated in every case once the appropriate range of chlorambucil concentrations for an individual subject's lymphocytes had been established.

The untreated patients showed a sensitivity to a concentration of chlorambucil of the same order of that achievable *in vivo* and similar to those used in direct cytotoxicity assays (Bosanquet *et al.*, 1983; Bosanquet, 1991). Exposure of the cells to chlorambucil was, however, continuous over a period of 4 days in the current assay. There is evidence (Kern & Weisenthal, 1990) that prolonged exposures to the cytotoxic agent at high concentrations are necessary in order to produce valid results in this type of assay.

The results of this small pilot study provide evidence of primary drug resistance in untreated patients; moreover the $ID_{50}$ values found in those patients in whom there was clinical evidence of chlorambucil resistance were higher than those of patients found to be sensitive to treatment without any overlap between the two groups. Furthermore the evolution of clinical resistance could be identified; in all but one instance the $ID_{50}$ increased with treatment and this was related to a fall in the slope of the dose:response curve. Increasing drug resistance is likely to result from selective elimination of the more sensitive tumour cells although the effect of conditioning of surviving individual cells by exposure to the drug cannot be excluded. In the present study *in vitro* chlorambucil resistance appeared to be irreversible although sufficiently long-term studies required to confirm this have not yet been undertaken. The refractory patients showed an increase in cellular resistance to chlorambucil which appears to be maintained *in vivo* for many months after withdrawal of treatment and into the terminal phase of the disease.

The present data do not provide any indication of the mechanism of drug resistance in these patients. Increased activity of a membrane efflux pump mechanism has been identified in some patients with leukaemia (Kuwazuru *et al.*, 1990), but this is not thought to be relevant in chlorambucil resistance. Bank *et al.* (1989) have shown an equally rapid cytoplasmic uptake of chlorambucil in chemotherapy-resistant and chemotherapy-sensitive CLL cells. Drug neutralisation by increased intracellular glutathione (GSH) levels (Suzukake *et al.*, 1983) has been suggested Silber *et al.* (1989) found no difference in the GSH content of CLL lymphocytes compared to that of normal lymphocytes, although they did find intracellular GSH levels unstable in CLL cells in the presence of chlorambucil. In contrast to the present finding, these workers found normal lymphocyte viability less sensitive to the effects of chlorambucil than were CLL lymphocytes. Johnston *et al.* (1990) demonstrated a correlation between DNA cross link formation by chlorambucil in CLL cells and the product of the intracellular GSH level and glutathione S-transferase activity, but the number of cross links formed was not related to clinical drug sensitivity in their patients. The importance of intracellular GSH in modulating chlorambucil sensitivity merits further investigation.

The current assay has demonstrated a means by which chlorambucil sensitivity in CLL may be predicted and may provide a useful tool for the investigation of resistance mechanisms, the efficacy of new therapeutic agents or the effect of response modifiers. It has demonstrated that there is primary chlorambucil resistance established in some of the leukaemic cells prior to drug exposure. Therefore conditioning mechanisms may not be important and treatment strategies should be directed to the elimination of these resistant subclones at the time of first therapeutic intervention; current suppressive treatment serves inevitably to generate more resistant disease probably by selection of cells with a greater capacity to resist the effects of chlorambucil.

We are grateful to Mrs M. Watkins for providing the secretarial support.

### References

BANK, B.B., KANGANIS, D., LIEBES, L.F. & SIBLER, R. (1989). Chlorambucil pharmacokinetics and DNA binding in chronic lymphocytic leukemia lymphocytes. *Cancer Res.*, **49**, 554.

BECH-HANSEN, N.T., SARANGI, F., SUTHERLAND, D.J.A. & LING, V. (1977). Rapid assays for evaluating the drug sensitivity of tumor cells. *J. Natl Cancer Inst.*, **59**, 21.

BINET, J.L., AUQUIER, A., DIGHIERO, G. & 16 others (1981). A new prognostic classification of chronic lymphocytic leukemia derived from a multivariate survival analysis. *Cancer*, **48**, 198.

BOSANQUET, A. (1985). Stability of solutions of antineoplastic agents during preparation and storage for *in vitro* assays. *Cancer Chemother. & Pharmacol.*, **14**, 83.

BOSANQUET, A. (1991). Correlations between therapeutic response of leukaemias and *in vitro* drug-sensitivity assay. *Lancet*, **337**, 711.

BOSANQUET, A.G., BIRD, M.C., PRICE, W.J.P. & GILBY, E.D. (1983). An assessment of a short-term tumour chemosensitivity assay in chronic lymphocytic leukaemia. *Br. J. Cancer*, **47**, 781.

FRENCH COOPERATIVE GROUP ON CHRONIC LYMPHOCYTIC LEUKAEMIA (1986). Effectiveness of 'CHOP' regimen in advanced untreated chronic lymphocytic leukemia. *Lancet*, **i**, 1346.

GALE, R.P. & FOON, K.A. (1985). Chronic lymphocytic leukaemia. Recent advances in biology and treatment. *Ann. Intern. Med.*, **103**, 101.

HANSON, J.A., BENTLEY, D.P., BEAN, E.A., NUTE, S.R. & MOORE, J.L. (1991). *In vitro* chemosensitivity testing in chronic lymphocytic leukaemia patients. *Leukaemia Res.*, **15**, 565.

ISAKOVIC, K. & LENERT, G. (1987). Reactivity of B leukemic lymphocytes of patients with chronic lymphocytic leukemia to PWM and PHA. *Blood Cells*, **12**, 355.

JOHNSTON, J.B., ISRAELS, L.G., GOLDENBERG, G.J. & 4 others (1990). Glutathione S-transferase activity, sulfhydryl group and glutathione levels and DNA cross-linking activity with chlorambucil in chronic lymphocytic leukemia. *J. Natl Cancer Inst.*, **9**, 776.

JOHNSTONE, A.P., MILLARD, R.E. & HUDSON, L. (1982). The roles of leukaemic and residual normal cells in the proliferative response of chronic lymphocytic leukaemic lymphocytes to mitogens. *Clin. Exp. Immunol.*, **47**, 689.

KERN, D.H. & WEINSENTHAL, L.M. (1990). Highly specific prediction of antineoplastic drug resistance with an *in vitro* assay using suprapharmacologic drug exposures. *J. Natl Cancer Inst.*, **82**, 582.

KUWAZURU, Y., YOSHIMURA, A., HANADA, S. & 7 others (1990). Expression of the multidrug transporter, P-glycoprotein, in acute leukaemia cells and correlation to clinical drug resistance. *Cancer*, **66**, 868.

MONTSERRAT, E., ALCALÁ, A., PARODY, R. & 10 others (1985). Treatment of chronic lymphocytic leukemia in advanced stages. *Cancer*, **56**, 2369.

ROBÈRT, K.-H., MÖLLER, E., GAHRTON, G., ERIKSSON, H. & NILSSON, B. (1978). B-cell activation of peripheral blood lymphocytes from patients with chronic lymphatic leukaemia. *Clin. & Exp. Immunol.*, **33**, 302.

SIENA, S., BREGNI, M., FORMOSA, A. & 5 others (1989). Immunotoxin-mediated inhibition of chronic lymphocytic leukemia cell proliferation in humans. *Cancer Res.*, **49**, 3328.

SILBER, R., POTMESIL, M. & BANK, B.B. (1989). Studies on drug resistance in chronic lymphocytic leukemia. *Adv. Enzyme Reg.*, **29**, 267.

SUZUZAKE, K., VISTICA, B.P. & VISTICA, D.T. (1983). Dechlorination of L-phenylalanine mustard by sensitive and resistant tumor cells and its relationship to intracellular glutathione content. *Biochem. Pharmacol.*, **32**, 165.

TWENTYMAN, P.R., FOX, N.E. & REES, J.K.H. (1989). Chemosensitivity testing of fresh leukaemia cells using the MTT colorimetric assay. *Br. J. Haematol.*, **71**, 19.

VEERMAN, A.J.P. & PIETERS, R. (1990). Drug sensitivity assays in leukaemia and lymphoma. *Br. J. Haematol.*, **74**, 381.

WEISENTHAL, L.M. (1981). *In vitro* assays in preclinical antineoplastic drug screening. *Sem. Oncol.*, **8**, 362.

WEISENTHAL, L.M., DILL, P.L., FINKLESTEIN, J.Z., DUARTE, T.E., BAKER, J.A. & MORAN, E.M. (1986). Laboratory detection of primary and acquired drug resistance in human lymphatic neoplasms. *Cancer Treatment Rep.*, **70**, 1283.